# Inefficient cognitive control in adult ADHD: evidence from trial-by-trial Stroop test and cued task switching performance

**DOI:** 10.1186/1744-9081-3-42

**Published:** 2007-08-20

**Authors:** Joseph A King, Michael Colla, Marcel Brass, Isabella Heuser, DY von Cramon

**Affiliations:** 1 Max Planck Institute for Human Cognitive and Brain Sciences, Department of Cognitive Neurology, Stephanstr. 1A, D-04103 Leipzig, Germany; 2 Charité-Universitätsmedizin Berlin, Campus Benjamin Franklin, Department of Psychiatry and Psychotherapy, Eschenallee 3, D-14050 Berlin, Germany; 3 Ghent University, Department of Experimental Psychology, Henri Dunantlaan 2, BE-9000 Ghent, Belgium

## Abstract

**Background:**

Contemporary neuropsychological models of ADHD implicate impaired cognitive control as contributing to disorder characteristic behavioral deficiencies and excesses; albeit to varying degrees. While the traditional view of ADHD postulates a core deficiency in cognitive control processes, alternative dual-process models emphasize the dynamic interplay of bottom-up driven factors such as activation, arousal, alerting, motivation, reward and temporal processing with top-down cognitive control. However, neuropsychological models of ADHD are child-based and have yet to undergo extensive empirical scrutiny with respect to their application to individuals with persistent symptoms in adulthood. Furthermore, few studies of adult ADHD samples have investigated two central cognitive control processes: interference control and task-set coordination. The current study employed experimental chronometric Stroop and task switching paradigms to investigate the efficiency of processes involved in interference control and task-set coordination in ADHD adults.

**Methods:**

22 adults diagnosed with persistent ADHD (17 males) and 22 matched healthy control subjects performed a manual trial-by-trial Stroop color-word test and a blocked explicitly cued task switching paradigm. Performance differences between neutral and incongruent trials of the Stroop task measured interference control. Task switching paradigm manipulations allowed for measurement of transient task-set updating, sustained task-set maintenance, preparatory mechanisms and interference control. Control analyses tested for the specificity of group × condition interactions.

**Results:**

Abnormal processing of task-irrelevant stimulus features was evident in ADHD group performance on both tasks. ADHD group interference effects on the task switching paradigm were found to be dependent on the time allotted to prepare for an upcoming task. Group differences in sustained task-set maintenance and transient task-set updating were also found to be dependent on experimental manipulation of task preparation processes. With the exception of Stroop task error rates, all analyses revealed generally slower and less accurate ADHD group response patterns.

**Conclusion:**

The current data obtained with experimental paradigms deliver novel evidence of inefficient interference control and task-set coordination in adults with persistent ADHD. However, all group differences observed in these central cognitive control processes were found to be partially dependent on atypical ADHD group task preparation mechanisms and/or response inconsistency. These deficiences may have contributed not only to inefficient cognitive control, but also generally slower and less accurate ADHD group performance. Given the inability to dissociate these impairments with the current data, it remains inconclusive as to whether ineffecient cognitive control in the clinical sample was due to top-down failure or bottom-up engagement thereof. To clarify this issue, future neuropsychological investigations are encouraged to employ tasks with significantly more trials and direct manipulations of bottom-up mechanisms with larger samples.

## Background

Attention-deficit/hyperactivity disorder (ADHD) is a chronic early-onset syndrome of developmentally inappropriate levels of inattention, hyperactivity, and impulsivity [1]. Although ADHD has traditionally been considered a childhood disorder, evidence from studies of clinical correlates, family history, treatment response and various laboratory measures confirm the validity of the diagnosis ADHD in adulthood [2]. While core symptoms are often clearly evident in overt disruptive behavior and learning problems in children with ADHD, adults with persisting symptoms typically display subtler cognitive and behavioral impairments which, nonetheless, are often associated with significant educational, occupational, interpersonal, emotional, and even legal difficulties [3,4]. In order to advance the knowledge of the brain-behavior relationships underlying the developmental course of ADHD, further neuropsychological data is needed; particularly from adults.

Contemporary neuropsychological models of ADHD implicate impaired cognitive control as contributing to disorder characteristic behavioral deficiencies and excesses; albeit to varying degrees [for reviews, see [5,6]]. According to the traditional neurocognitive view of ADHD, a core deficiency in cognitive control processes subserving the orchestration of thought and action with internal goals arises from neurodevelopmental anomalies in the prefrontal cortex [7]. This model postulates that ADHD-type cognitive control deficits should be particularly manifest in aberrant (1) inhibition of inadequate but prepotent responses, (2) stopping of ongoing responses and (3) interference control. Alternative "dual-process" models of ADHD [8-14] emphasize the dynamic interplay of bottom-up driven factors such as activation, arousal, alerting, motivation, reward and temporal processing with top-down cognitive control processes in explaining the well-replicated findings of ADHD sample impairment on tasks tapping executive functions (for a recent meta-analysis, see [15]). According to dual-process models, impaired ADHD group executive task performance is, at least partially, due to disturbances in bottom-up basal ganglia- and/or cerebellar-thalamo-cortical loops subserving the regulation of cognitive state.

Nonetheless, neuropsychological models of ADHD are child-based and have yet to undergo extensive empirical scrutiny with respect to their application to individuals with persistent symptoms in adulthood. Although a number of recent adult ADHD studies have targeted various component cognitive control processes with experimental chronometric paradigms [e.g. [16-26]], few behavioral studies have investigated interference control or task-set coordination in ADHD with the precise measurement afforded by computerized tasks. The current study investigated the efficiency of cognitive control processes involved in interference control and the flexible coordination of multiple task-sets in adults with persistent ADHD by employing two experimental measures with a clinical sample and healthy control subjects; a manual trial-by-trial Stroop color-word test and an explicitly cued task switching paradigm. Modified versions of the employed tasks have been demonstrated to be particularly sensitive to lateral prefrontal cortex function in healthy individuals (see e.g. [27]; a brain region implicated both structurally [28-31] and functionally [32-35] in ADHD (for reviews, see [36-39]).

The Stroop test [40] is arguably the benchmark laboratory measure of cognitive control. The critical measure of Stroop tests, the so-called "Stroop effect", refers to the robust performance decrement in response to stimuli which contain a dominantly represented task-irrelevant dimension versus those which contain less strongly represented task-relevant or neutral features [41]. Previous studies of ADHD samples with Stroop tasks have delivered mixed results leading to mixed interpretations. Indicative of disrupted interference control over task-irrelevant stimulus features in ADHD, two recent meta-analyses of neuropsychological functioning in adult ADHD [42,43] and three recent meta-analyses of Stroop performance in child and young adult ADHD samples [44-46] all found moderate to large group effect sizes for the incongruent color-word measure of standardized clinical Stroop tests (e.g[47]). However, indicative of generalized slowing in ADHD on Stroop tests, all cited meta-analyses also reported that when the interference score is calculated by subtracting performance on the neutral color condition from that on the incongruent color-word measure, effect sizes are smaller than those found for the respective baseline measures. Furthermore, all studies incorporated in the cited meta-analyses employed paper and pencil versions of the Stroop test which do not allow for fine-grained performance analysis (cf. [48]). To our knowledge, only two studies of adults with persisting ADHD have employed chronometric Stroop tasks [49,50]. Nevertheless, both studies employed paradigms in which Stroop conditions are presented in separate blocks. We expected that the employed trial-by-trial Stroop paradigm would be more sensitive to detecting behavioral group differences in interference control.

Task switching paradigms require the performance of two simple discrimination tasks, such as determining whether a number is even or odd or whether a letter is a consonant or a vowel. Depending on which task is to be performed in a given trial, subjects are required to attend to and classify different stimulus features. Switching between tasks is usually associated with a sizeable performance decrement in comparison to that when the same task is repeated. In order to switch tasks efficiently, a previously active task-set must be inhibited in favor of the alternative task-set. Two types of switch-related performance decrements are readily observable in blocked explicitly-cued task switching paradigms: switch costs and mixing costs. While switch costs (i.e. switch *trial *– repetition *trial *performance) are assumed to index transient cognitive control processes involved in task-set updating, mixing costs (i.e. switch *block *repetition trial – pure repetition *block *trail performance) are thought to reflect a more sustained component, such as increased maintenance demands associated with keeping multiple task-sets at a relatively high level of activation (e.g. [51]; for an opposing view, see [52]; for a general review on task switching, see [53])

Different aspects of task switching efficiency can be investigated by manipulating the delay between a cue indicating which task is to be performed and the presentation of the stimulus (cue-task interval or CTI) or by manipulating the relevancy of stimulus features (stimulus valence). Studies using task-cueing paradigms have revealed that performance costs are significantly reduced if subjects are allowed to prepare for an upcoming task via experimental variation of the CTI [54,55]. Studies manipulating stimulus valence have revealed that performance costs are also significantly reduced if the current stimulus contains only one task-relevant dimension (i.e. is univalent) versus those containing attributes relevant for both possible tasks (i.e. are bivalent) [56]. By incorporating these manipulations in the employed task switching paradigm, we were not only able to investigate the efficiency of transient task-set updating and sustained task-set maintenance in ADHD adults, but also the influence of task preparation and interference on cognitive flexibility.

Despite the advantages of task switching paradigms in measuring processes pertinent to cognitive flexibility (e.g. response latency *and *error rate data, measurement of *multiple *distinct processes), most ADHD studies have employed the Wisconsin Card Sorting Test (WCST). Inconsistent findings of group differences in WCST response perseveration rates are reflected in low effect sizes found by recent meta-analyses [15,42]. To our knowledge, only two studies have investigated WCST-like task-set shifting in ADHD with explicitly cued task switching paradigms. Cepeda et al. [57] and Kramer et al. [58] found unmedicated ADHD children to produce larger switch costs in comparison to methylphenidate medicated ADHD children and healthy control subjects. Importantly, suggestive of intact sustained task-set maintenance in childhood ADHD, neither study found group differences in mixing costs. Despite differences in the employed task switching paradigms, the integration of the results of the current study with previous findings of childhood ADHD group task switching deficiencies should contribute needed information regarding the possible developmental trajectory of cognitive flexibility in this patient population.

To summarize, ADHD is currently conceptualized as neurocognitive disorder characterized by impairment in cognitive control processes. However, despite the expectations of currently accepted child-based neuropsychological models of ADHD, studies investigating the cognitive control components of interference control and task-set coordination with traditional measures have generally delivered inconsistent results. Furthermore, the investigation of these processes in adults with persistent child-onset ADHD has been largely neglected. We expected that the employed chronometric trial-by-trial Stroop test would detect group differences specific to interference control efficiency. We hypothesized that group differences on the task switching paradigm in transient task-set updating (i.e. switch costs) and sustained task-set maintenance (i.e. mixing costs) would be further evident as a function of task preparation (i.e. CTI manipulations) and interference (i.e. valence manipulations). Results should deliver information relevant to the neurocognitive modelling of ADHD across the lifespan.

## Methods

### Subjects

The two participant groups included in the study sample comprised a total of 44 individuals aged between 18–45 years old. All subjects provided written consent in accordance to the guidelines of the Charité University Medicine ethics commission after being informed about study procedures. The experimental group consisted of 22 self- and clinic referred patients (17 males) diagnosed with ADHD. The community control group included 22 volunteers (17 males) recruited via newspaper advertisements.

Clinical assessment of patient participants was conducted according to the diagnostic guidelines for ADHD in adulthood as outlined by an expert consensus of the German Society for Psychiatry, Psychotherapy and Neurology [59]. The cornerstone of the employed diagnostic protocol was the semi-structured Conners' Adult ADHD Diagnostic Interview for DSM-IV (CAADID). Several standardized self-report and collateral informant rating scales designed to quantify ADHD symptoms both currently and retrospectively were also employed. These included the Connors Adult ADHD Rating Scales (CAARS) [60], the Wender-Utah-Rating-Scale-German-Shortform (WURS-k) [61] and the adult ADHD Checklist (ADHD-CL) [62]. Relevant archival data (i.e. school and occupational records) were reviewed in every case. Informant interviews were conducted in 17 of 22 cases. Standardized screening for comorbidities was performed by using the Symptom Checklist -90R [63]. A diagnosis was given to individuals fulfilling DSM-IV criteria for childhood ADHD only under consensus of a graduate level clinical psychologist/psychiatrist in training and a board certified psychiatrist (M.C.) after careful review of the data acquired via this assessment protocol.

A total of 36 patients referred to the Charité University Medicine Campus Benjamin Franklin Adult ADHD Outpatient Clinic performed the experimental neuropsychological battery comprised of the Stroop test and the task switching paradigm. Patients determined to have persisting childhood-onset ADHD, regardless of subtype, were included in the experimental group. They were excluded from the study sample if they currently evidenced more significantly impairing DSM-IV Axis I diagnoses, current neurological impairment, had a history suggestive of psychosis, had a history of neurological disorder, were currently receiving psychoactive medication (with the exception of methylphenidate, in which case a 48 hour washout period was mandatory; n = 2), were older than 45 years of age or their estimated verbal IQ was < 90. 14 participants (8 male, 6 female) did not meet inclusion criteria. Evidence for childhood-onset ADHD was not substantiated for one female and four male participants.

Potential control subjects were briefly screened for psychiatric and neurological histories. Individuals reporting no such history were selected to match the ADHD group as closely as possible for age, sex, and education level. Invited volunteers were briefly interviewed and physically examined for neurological impairment. Volunteers reporting a history of contact with psychiatric/neurological services, evidencing a history indicative of a neuropsychiatric disorder, exhibiting gross neurological impairment, currently receiving a regime of any psychoactive medication or having an estimated verbal IQ < 90 were not included in the control group. A total of 24 volunteers completed the experimental test battery. 2 male volunteers did not meet inclusion criteria.

Sample demographics and estimates of intellectual functioning are summarized in Table 1. The mean age of the ADHD group did not differ from that of the control group (*t*(42) = .02; *n.s*.). The mean education level as calculated by the years normally required to complete a given type of formal education in Germany did not differ between the groups (*t*(42) = .24; *n.s*.). In addition to controlling for group differences with regard to age, sex and education level, commonly employed German standardized estimate measures of verbal [64] and figural intelligence [65] were administered to all participants.The mean estimated verbal IQ of the ADHD group did not differ from that of the control group (*t*(42) = 1.0; *n.s*.). Likewise, the mean estimated figural IQ of the ADHD group did not differ from that of the control group (*t*(42) = 1.3; *n.s*.). Clinical data obtained from patient participants with two of the employed retrospective (WURS-k) and current (ADHD-CL) symptom screening scales are also summarized in Table 1. Symptom severity in the current ADHD sample as measured by these scales is comparable with the values obtained in a validation study of the abridged version of the German WURS [66], as well as a recent German study employing the ADHD-CL [67]

**Table 1:** Demographic characteristics, intellectual functioning estimates and ADHD-specific self-rating data

	**ADHD (n = 22)**	**control (n = 22)**
**gender**		
(M/F)	17/5	17/5
**demographic data**		
Age (years)	30.0 (8.1)	30.1 (7.2)
education level (years)	14.4 (1.9)	14.5 (1.8)
**intellectual functioning estimates**		
verbal IQ (MWT-B)	106.5 (7.3)	108.9 (8.3)
figural IQ (LPS-3)	107.5 (8.7)	110.6 (6.7)
**ADHD self-rating scales**		
retrospective (WURS-k)	26.2 (6.3)	not tested
current (ADHD-CL)	27.2 (3.9)	not tested

### Procedures and Apparatus

All subjects were run in the neuropsychology laboratory at the Charité University Medicine Campus Benjamin Franklin Clinic for Psychiatry and Psychotherapy. ADHD subjects were tested within the framework of the clinical assessment protocol. Control group subjects were tested immediately following a psychiatric interview, neurological examination and IQ estimation. Before performing the test battery, participants were verbally provided with the general instructions, procedures and rules of the testing session in a standardized manner. The tasks were administered across subjects in a counterbalanced sequence. Participants received written instructions for each of the experiments immediately prior to performing practice trials for the respective tests.

Experiments were run on an IBM compatible PC (Intel Pentium Processor, 32 MB RAM) with a 235 × 170 mm monitor (Samsung SyncMaster 700(M)s SGA with 60.3 Hz). In order to allow for easy discrimination of the stimuli, participants sat approximately 60 cm from the monitor. Responses were made by means of left and right index finger button presses on a custom built external response keypad placed on a level surface between the subject and the monitor. Responses were registered by means of the Experimental Run Time System (ERTS, BeriSoft Corporation) and Exkey Keyboard Logic (EXKEY, BeriSoft Corporation).

### Tasks

#### Stroop Test

An experimental manual trial-by-trial version of the Stroop test was employed. Stimuli were German language color words and letter strings presented centrally on a computer monitor in the colors red, green, blue and yellow against a gray background. Participants were instructed to indicate the color in which stimuli were presented by means of a button press. In order to minimize working memory load, two-letter abbreviations of the German language color names („ro", „gr", „bl", and „ge") indicating the correct mapping of the colors to the buttons of the response keypad appeared simultaneously with the stimulus presentation in white. There were three experimental conditions: In the neutral baseline condition, „XXXX" was presented in one of the four possible colors. In the congruent condition, color words were presented in a semantically analog color. In the incongruent condition, color words were presented in a color incompatible with the meaning of the written color word. Participants responded by pressing one of the buttons on the keypad which were each mapped to a specific color (button 1 = red, button 2 = green, button 3 = blue, button 4 = yellow).

Each trial began with the presentation of a white fixation cross in the middle of the computer screen (200 ms). Target stimuli were presented following fixation (1500 ms). The screen went blank following stimulus presentation (500 ms). The maximum response time was 2000 ms after the initial presentation of the target stimulus. Immediately following the maximum response window, verbal feedback informing participants whether their response was correct, incorrect or too slow was presented (200 ms). Following feedback presentation, the screen went blank until the beginning of the next trial (2000 ms).

**Figure 1:**
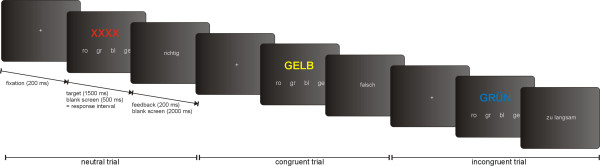
**Manual trial-by-trial Stroop color-word test**. Trial temporal structure with examples of the three possible trial types.

Participants performed 20 experimenter-paced practice trials. The experimental session included 96 trails (32 baseline, 32 congruent, 32 incongruent). Trial order and stimulus color were pseudorandomized to equate transition probabilities between trial types and the target color frequency under the three experimental conditions. The total task duration was approximately 9 minutes. Examples of each experimental condition and the Stroop task trial temporal structure are depicted in Fig. 1.

#### Task Switching

In each trial of the blocked explicitly cued task switching paradigm, colored shapes were presented centrally on the monitor as target stimuli against a light gray background. Participants had to perform one of two possible tasks in each trial: the "color" task or the "shape" task. The task to be performed in a given trial was indicated by a verbal cue printed in German in the upper portion of the screen. In color task trials, participants had to decide whether a presented shape was red or blue by means of a button press. In shape task trials, they had to classify the presented shape as a square or diamond. Correct responses were made by means of a left button press for a red target stimulus in color trials and a square in shape trials. Correct responses were made by means of a right button press for a blue target stimulus in color trials and a diamond in shape trials. The paradigm consisted of two types of task blocks: single task blocks (pure repetition blocks; PRB) and mixed task blocks (MB). In PRBs, participants had to repeatedly perform exclusively either the color task (C block) or the shape task (S block). In MBs, participants had to alternate in an unpredictable manner between performance of both of the tasks (M block). MBs included both repetition and switch trials. Two types of stimulus valence were used: univalent stimuli and bivalent stimuli. A univalent stimulus included one characteristic, which was relevant for one of the two tasks and another characteristic which was irrelevant for both tasks (e.g. a red ellipse in a color trial; a yellow diamond in a shape trial). A bivalent stimulus included characteristics which were relevant for both tasks (e.g. a red square; a blue diamond).

Each trial began with the presentation of a white fixation cross in the center of the screen (100 ms). Following fixation, depending on the experimental variation of the CTI, the screen went blank for either 1100 ms (in short CTI trials) or 200 ms (in long CTI trials). In short CTI trials, cues were presented 100 ms prior to stimulus presentation. In long CTI trials, cues were presented 1000 ms prior to stimulus presentation. Cues were the German words for "color" and "shape" presented in white directly above target stimuli. Target stimuli were presented up to the maximum response time of 2000 ms. If a response was made within this timeframe, verbal feedback informing the participants whether their response was correct or incorrect was instantaneously displayed (500 ms). If no response was made within the maximum response window, feedback informing the participants that their response was too slow was displayed. Following feedback presentation, the screen went blank until the beginning of the next trial (500 ms).

Participants performed 15 practice trials of each of the three types of task blocks. The practice session always began with one of the two single task blocks which were administered across participants in a counterbalanced manner. Practice session trial timing was identical to that in the experimental session. The experimental session was comprised of 12 task blocks; each including 24 trials. There were two of each of the single task blocks and eight mixed task blocks. Two fixed block sequences were used:

• block sequence 1: M S M M C M M S M M C M

• block sequence 2: M C M M S M M C M M S M

**Figure 2:**
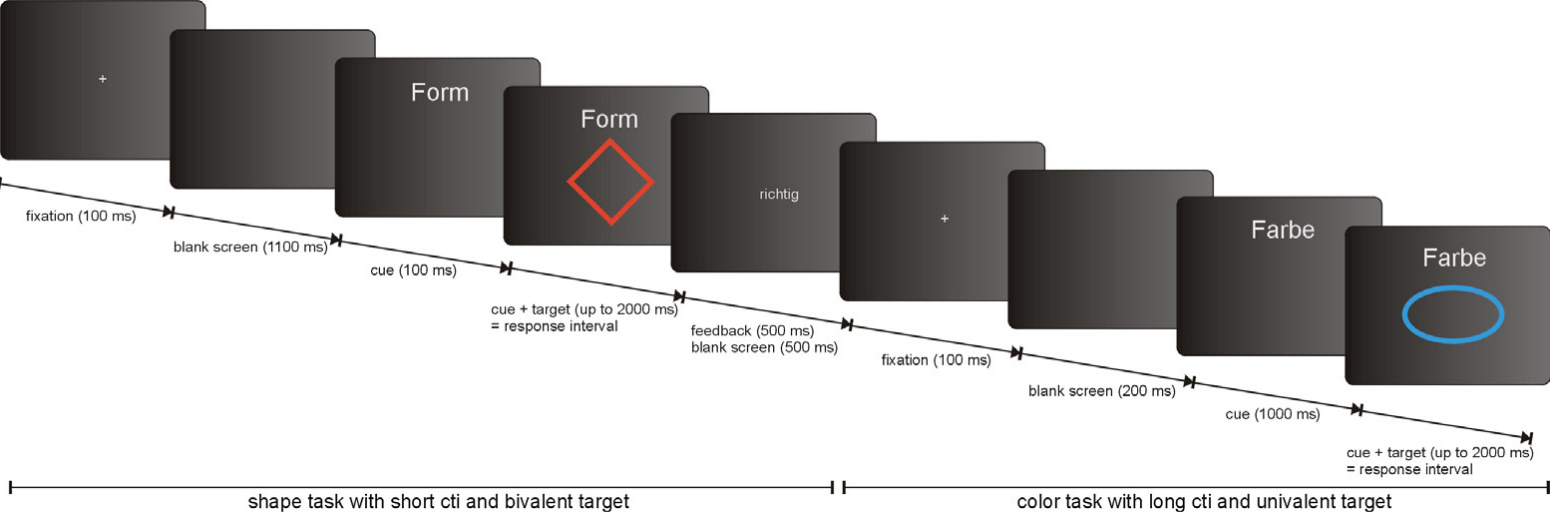
**Blocked explicitly cued task switching paradigm**. Trial temporal structure with examples of the two possible trial types respectively with manipulations of CTI and stimulus valence.

Block sequence 1 was administered to participants who performed the S block first during the practice session. Block sequence 2 was administered to participants who performed the C block first during the practice session. Each block began with an instruction informing the participant which block was to be performed in the following trials. Trials were presented in a pseudorandomized manner, in order to assure an equivalent number of trial types, trial type transitions, as well as with regard to stimulus valence and CTI variation. The total task duration was approximately 16 minutes. Examples of each experimental manipulation and the task switching trial temporal structure are depicted in Fig. 2.

#### Design and Analysis

In a within-subject design, performance on the Stroop test and the task switching paradigm was investigated. Group (ADHD vs. control) was a between-subject variable in all analyses. The independent variable of the Stroop test was stimulus congruency (neutral, congruent, incongruent). The independent variables of the task switching analyses were trial type (repetition, switch), block type (PRB, MB), CTI (100 ms, 1000 ms) and stimulus valence (univalent, bivalent). The dependent variables for both tasks were reaction time (RT) and error rate. Incorrect responses (i.e. overt errors), response failures (i.e. RT > 2000 ms) and premature responses (i.e. RT < 100 ms; regardless of accuracy) were excluded a priori from RT data analyses. The first trial of each block of the task switching paradigm was also discarded. Identical to the analysis method employed in the only known trial-by-trial Stroop investigation of an ADHD sample [68], Stroop test data were subjected to 2 separate sets of repeated-measures ANOVAs which respectively investigated group differences in Stroop interference (i.e. incongruent vs. neutral) and facilitation (i.e. congruent vs. neutral). Identical to the data analytic strategy employed in the only known cued task switching studies of ADHD samples, [57,58], task switching data were subjected to two separate sets of repeated-measures ANOVAs which respectively tested group differences in switch costs (i.e. switch – repetition trials in MBs) and mixing costs (i.e. MB repetition trials – PRB trials). A supplementary analysis of the task switching data restricted to PRBs explored whether the groups differed according to CTI and valence manipulations in block contexts not requiring task-set coordination. Statistical analyses were conducted using the Statistical Package for the Social Sciences 15.0 for Windows (SPSS Inc.) at an alpha threshold of .05 (two-tailed). Significant condition × group interactions obtained with the task switching paradigm were further analyzed with appropriate *t*-tests while adjusting significance thresholds for multiple comparisons according to Bonferroni. Effect sizes are reported as partial Eta squared for theoretically relevant effects of the group factor. Detailed data reporting is limited to findings confirming that task manipulations were successful and those of particular theoretical relevance for neurocognitive modelling of ADHD.

## Results

### Experiment I: Manual Stroop Task

Table 2 presents the mean RTs and standard errors of the mean for correct responses and error rate percentages for both groups under each experimental condition. Table 3 presents a summary of all results obtained from the Stroop test.

**Table 2:** Stroop test performance data. Mean response latencies and error rate percentages for ADHD and control groups under all conditions of the Stroop test (standard errors of the mean shown in parentheses).

	**ADHD (n = 22)**	**control (n = 22)**
**condition**	RT (ms) errors (%)	RT (ms) errors (%)
neutral	880 (37) 4.8 (1.9)	772 (30) 3.6 (1.3)
congruent	863 (41) 4.3 (1.5)	741 (32) 2.3 (0.7)
incongruent	999 (45) 9.9 (2.0)	841 (27) 5.0 (1.6)

**Table 3:** Stroop test ANOVAs. Summary of significant effects obtained from 4 ANOVAs conducted with Stroop test data. indicates *p *< .05. ** indicates *p *< .01. *** indicates *p *< .001. Bold print indicates significant group × condition interactions. Detailed data reporting in text.

**interference**	2 (group) × 2 (stimulus congruency; incongruent vs. neutral)
*RT*	
	group**
	stimulus congruency***
	group × stimulus congruency*
*error rate*	
	stimulus congruency***
	group × stimulus congruency*

**facilitation**	2 (group) × 2 (stimulus congruency; congruent vs. neutral)

*RT*	
	group*
	stimulus congruency***
*error rate*	
	no significant effects

### Stroop Interference

**Figure 3:**
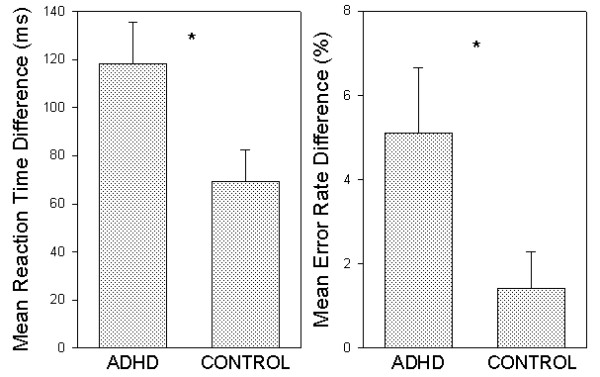
**Significant group × Sroop interference interactions**. Mean reaction time (left) and error rate percentage (right) differences between incongruent and neutral conditions (i.e. Stroop interference) of the Stroop test for ADHD and control groups. * indicates *p *< .05. Bars represent standard errors of the mean.

A 2 (group) × 2 (trial type; neutral vs. incongruent) ANOVA of the Stroop test response latency data investigated whether the groups differed as a function of Stroop interference. Significant main effects for both factors and their interaction were obtained. The expected main effect of trial type (*F*(1, 42) = 74.8; *p *< .001) indicated that the Stroop effect was generally produced by the experimental manipulation. The main effect of group (*F*(1, 42) = 7.4; *p *< .01; η^2 ^= .150) demonstrated that the ADHD group produced generally slower responses on the contrasted measures. The hypothesized group × trial type interaction (*F*(1, 42) = 5.1; *p *< .05; η^2 ^= .108) revealed that the ADHD group displayed more sensitivity to the interfering effect of dominantly represented task-irrelevant verbal stimulus features than control subjects (see Fig. 3).

The corresponding performance accuracy analysis reconfirmed the success of the experimental manipulation by disclosing a significant main effect for trial type (*F*(1, 42) = 13.6; *p *< .001). The Stroop effect was evident in the error rates of both groups. Although the groups were not found to differ generally in error rates on the contrasted trial types (*F*(1, 42) = 1.9; n.s.), a group discrepancy was again evident in the critical trial type × group interaction (*F*(1, 42) = 4.3; *p *< .05; η^2 ^= .094). Indicative of disrupted interference control in adult ADHD, patient subjects' accuracy on incongruent – neutral Stroop test conditions significantly differed from that of the control group (see again, Fig. 3).

### Stroop Facilitation

Group differences resulting from the facilitative effect of color-word congruency were tested by comparing performance on congruent and neutral trials. Indicating that RTs were generally faster on congruent trials, the trial type factor reached significance (*F*(1, 42) = 9.2; *p *< .005). Importantly, the group factor also reached significance (*F*(1, 42) = 5.5; *p *< .05; η^2 ^= .116). ADHD subjects produced generally slower responses on trials not comprised of distracting verbal information. However, demonstrating that the speeding of responses to ink color-congruent color words in comparison to neutral stimuli was equivalent between the groups, no interaction of trial type and group was found (*F*(1, 42) = .74; n.s.).

The analog analysis of the accuracy data revealed no variation in error rates attributable to trial type either generally (*F*(1, 42) = 1.3; n.s.) or between the groups (*F*(1, 42) = .19; n.s.). Response accuracy was not facilitated by stimulus congruency. Furthermore, the groups were not found to generally differ in response accuracy on the contrasted trial types (*F*(1, 42) = .81; n.s.).

### Experiment II: Task switching

Tables 4 and 5 respectively present the mean RTs and standard errors of the mean for correct responses and error rate percentages for both groups under each of the experimental conditions of the task switching paradigm. Table 6 presents a summary of all significant main effects and group × condition interactions obtained from the task switching paradigm.

**Table 4:** Task switching reaction time data. Mean reaction times for ADHD and control groups under each of the task switching trial configurations (standard errors of the mean shown in parentheses).

			trial type		
			
group	CTI	valence	repetition (PRB)	repetition (MB)	switch (MB)
**ADHD**	1000 ms	univalent	507 (28)	567 (37)	611 (40)
		bivalent	519 (30)	563 (35)	600 (35)
	100 ms	univalent	575 (27)	755 (42)	829 (45)
		bivalent	617 (35)	795 (42)	819 (43)

**control**	1000 ms	univalent	399 (14)	444 (17)	503 (26)
		bivalent	425 (16)	467 (21)	507 (23)
	100 ms	univalent	474 (16)	631 (22)	721 (32)
		bivalent	468 (17)	690 (27)	740 (31)

**Table 5:** Task switching response accuracy data. Mean error rate percentages for ADHD and control groups under each of the task switching trial configurations (standard errors of the mean shown in parentheses)

			trial type		
			
group	CTI	valence	repetition (PRB)	repetition (MB)	Switch (MB)
**ADHD**	1000 ms	univalent	6.1 (1.3)	4.1 (1.0)	8.4 (2.1)
		bivalent	7.0 (1.8)	8.3 (1.8)	11.2 (2.1)
	100 ms	univalent	4.6 (1.2)	6.4 (1.9)	9.7 (1.9)
		bivalent	5.9 (1.7)	11.2 (2.0)	13.4 (2.1)

**control**	1000 ms	univalent	1.9 (0.8)	1.4 (0.6)	3.1 (0.6)
		bivalent	2.2 (0.6)	6.1 (1.3)	4.0 (1.0)
	100 ms	univalent	3.0 (1.0)	1.2 (0.5)	5.4 (1.2)
		bivalent	2.5 (0.7)	3.3 (1.1)	9.0 (1.6)

### Switch costs

ANOVAs of the performance data from MBs with the group factor (ADHD vs. control) × trial type (repetition vs. switch) × CTI (100 ms vs. 1000 ms) × valence (univalent vs. bivalent) investigated group differences in the efficiency of transient task-set reconfiguration. The CTI manipulation served to measure how switching efficiency varies depending on the length subjects are allotted to prepare for an upcoming task. The valence manipulation served to measure how interference influences switching efficiency. Confirming that the task manipulations were associated with the expected changes in RTs, significant main effects were obtained for the factors of trial type (*F*(1, 42) = 60.0; *p *< .001), CTI (*F*(1, 42) = 358.5; *p *< .001), and valence (*F*(1, 42) = 4.4; *p *< .05). Responses were generally slower on switch trials than on repetition trials. RTs profited generally from a longer preparation period (i.e. CTI) and from trials comprised of stimuli features relevant only for the current task (i.e. univalent stimuli). A significant main effect of group was evident (*F*(1, 42)= 5.8; *p *< .05; η^2 ^= .121). Independent of task manipulations, ADHD participants produced slower responses than the control group in MBs. However, none of the experimental factors or factor interactions interacted with the group factor in the RT data (all *F*s < 2.3). Contrary to our hypotheses, the groups did not differ in switching efficiency as measured by RTs.

**Table 6:** Task switching ANOVAs. Summary of significant main effects and group × condition interactions obtained from 6 ANOVAs conducted with task switching data. ^+ ^indicates *p *= .07 * indicates *p *< .05. ** indicates *p *< .01. *** indicates *p *< .001. Bold print indicates significant group × condition interactions. Detailed data reporting in text.

**switch cost ANOVAs**	2 (group) × 2 (MB trial type) × 2 (CTI) × 2 (valence)
*RT*	
	group*
	
	leftMB trial type ***
	leftvalence*
*error rate*	
	group***
	MB trial type***
	CTI*
	valence***
	group × MB trial type × CTI**

**mixing cost ANOVAs**	

*RT*	
	group***
	block type***
	valence***
	group × block type × valence*
*error rate*	
	group***
	block type^+^
	valence***
	group × block type × CTI***

**pure repetition block ANOVAs**	2 (group) × 2 (CTI) × valence

*RT*	
	group***
	CTI***
	valence***
	group × block type × valence*
*error rate*	
	group*
	**group × CTI^+^**

**Figure 4:**
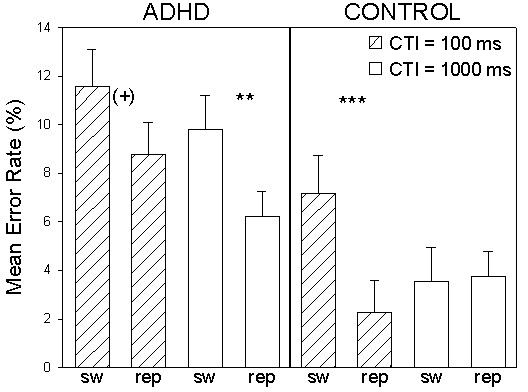
**Significant group × trial type × CTI interaction**. Mean error rates for the ADHD and control groups on MB switch and repetition trials as a function of CTI. (+) indicates *p *= .07. * indicates *p *= .01. *** indicates *p *< .001. Bars represent standard errors of the mean.

Indicating that MB task manipulations were also associated with a general variation in error rates, subjects were more accurate on repetition trials (*F*(1, 42) = 14.4; *p *< .001), on long CTI trials (*F*(1, 42) = 7.0; *p *<.05) and on trials containing univalent stimuli (*F*(1, 42) = 41.1; *p *< .001). Nonetheless, group did not interact directly with any of the within-subjects factors (all *F*s < .94). A significant main effect was again revealed for group (*F*(1, 42)= 10.0; *p *< .01; η^2 ^= .193). Independent of task manipulations, ADHD subjects had elevated error rate in MBs. Trial type interacted with CTI (*F*(1, 42) = 4.6; *p *< .05). Switch costs were generally reduced on long CTI trials. More importantly, a significant three-way group × trial type × CTI interaction was revealed (*F*(1, 42) = 8.7; *p *< .01; η^2 ^= .172). In order to investigate this interaction, paired *t*-tests compared within-group switch costs as a function of CTI variation. After adjusting the significance threshold for multiple comparisons, a trend toward switch costs in ADHD group error rates on short CTI trials was no longer clearly evident (*t*(21) = 1.9; *p *= .07). However, ADHD group switch costs were robust on long CTI trials (*t*(21) = 2.8; *p *= .01; corrected). In contrast, significant control group short CTI trial switch costs (*t*(21) = 4.3; *p *< .001; corrected) were eliminated by a longer preparation period (*t*(21) = .05; *p *= .9) (see Fig. 4). Relative to the control group, elevated ADHD group response inaccuracy on short CTI MB trials was not improved by an extended task preparation period. The remaining within-subjects factors did not interact with each other (all *F*s < 2.4) or with the group factor (all *F*s < 1.9).

### Mixing Costs

**Figure 5:**
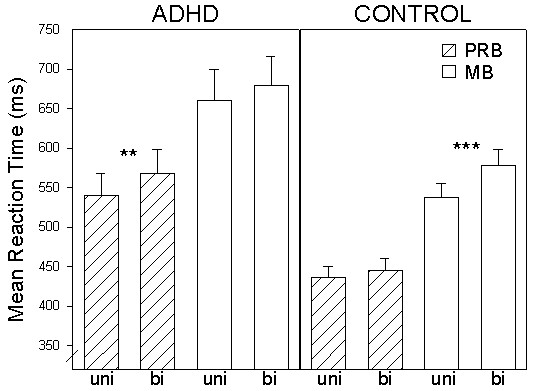
**Significant group × block type × valence interaction**. Mean reaction times for the ADHD and control groups on PRB and MB repetition trials as a function of stimulus valence. ** indicates *p *= .007. *** indicates *p *< .001. Bars represent standard errors of the mean.

In order to investigate group differences in factors involved in sustained task-set maintenance, ANOVAs contrasted group performance on repetition trials in task blocks in which task switches unpredictably occur (i.e. MBs) with that in task blocks requiring the repeated performance of a singular task (i.e. PRBs). Again, the influence of advance preparation (i.e. CTI manipulation) and interference (i.e. valence manipulation) was also explored. Significant main effects of block type (*F *(1, 42) = 161.6; *p *< .001), CTI (*F *(1, 42) = 256.3; *p *< .001) and valence (*F *(1, 42) = 16.9; *p *< .001) indicated that task manipulations produced the expected general variation of RTs. RTs on repetition trials in block contexts where switches unpredictably occur (MBs) were slower than those in blocks comprised a single task. Responses were generally slower on short CTI and bivalent repetition trials. Again, a main effect of group was found in the RT data (*F *(1, 42) = 9.8; *p *< .01; η^2 ^= .189). Independent of block context, ADHD subjects produced generally slower responses than control participants on repetition trials. However, group did not interact directly with any of the within-subjects factors (all *F*s < .69) in the RT analysis. Nonetheless, a three-way group × block type × valence interaction was revealed (*F *(1, 42) = 4.9; *p *< .05; η^2 ^= .105). In order to further investigate this interaction, paired *t*-tests compared within-group RTs for the valence manipulation on repetition trials separately in PRBs and MBs. While control group RTs were unaffected by the valence manipulation in PRBs (*t*(21) = 1.2; *p *= .24), they were significantly faster for MB repetition trials containing univalent vs. bivalent stimuli (*t*(21) = 5.8; *p *< .001; corrected). In contrast, ADHD group RTs were significantly slower on PRB bivalent vs. univalent trials (*t*(21) = 3.0; *p *= .007; corrected), but unaffected by stimulus valence in MBs (*t*(21) = 1.2; *p *= .26). (see Fig. 5). This unhypothesized finding revealed by the mixing cost analysis (but is not a mixing cost) provides further evidence for abnormal processing of task-irrelevant information in adult ADHD.

**Figure 6:**
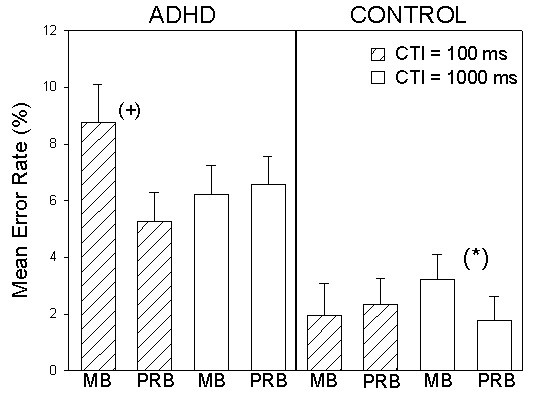
**Significant group × block type × CTI interaction**. Mean error rates for the ADHD and control groups on MB and PRB repetition trials as a function of CTI. (+) indicates *p *= .027. (*) indicates *p *= .017. Bars represent standard errors of the mean.

The analog error rate analysis revealed a trend towards generally decreased accuracy on repetition trials in MBs vs. PRBs (*F *(1, 42) = 3.7.; *p *= .06). A significant main effect for the valence factor (*F *(1, 42) = 18.7; *p *< .001) was also evident, but not for the CTI manipulation (*F *(1, 42) = .04; n.s.). While participants were less accurate on repetition trials containing stimulus features relevant for both possible tasks, accuracy did not generally vary according to the amount of time allotted to prepare for the upcoming task. Group did not interact directly with any of the within-subjects factors (all *F*s < .94). A main effect was again revealed for group (*F *(1, 42) = 9,6; *p *< .01; η^2 ^= .187). Independent from block context, the ADHD group committed more errors than control subjects on non-switch trials. Importantly, the groups were also found to differ as a function of a block type × CTI interaction (*F *(1, 42) = 16.0; *p *< .001; η^2 ^= .275). Paired *t*-tests comparing within-group mixing costs as a function of CTI variation investigated this interaction. Following significance threshold correction, ADHD group mixing costs in short CTI repetition trials (*t*(21) = 2.4; *p *= .027; corrected) were just shy of reaching significance. This mixing cost trend was no longer evident when cues were presented 1000 ms before stimulus presentation (*t*(21) = .43; *p *=.67). In contrast, mixing costs were not evident in control group error rates on short CTI repetition trials (*t*(21) = .71; *p *=.49), but just shy of significance in long CTI repetition trials (*t*(21) = 2.6; *p *= .017; corrected) (see Fig. 6). The group × block type × CTI interaction can likely be attributed to at least two factors: 1) ADHD group elimination of short CTI mixing costs on long CTI trials 2). a floor effect in control group repetition trial error rates. Regardless of the atypical control group response pattern, an elongated preparation interval evidently aided ADHD subjects in maintaining task-set in block contexts demanding flexible coordination of two task-sets. None of the remaining within-group factors interacted with the group factor (all *F*s < 1.1).

### Pure repetition blocks

In order to investigate whether group performance differed as a function of CTI and valence manipulations in block contexts not requiring task-set maintenance or updating, supplementary analyses were restricted to PRBs. A 2 (group) × 2 (CTI) × 2 (valence) ANOVA of the PRB response latency data revealed significant main effects for both within-subjects factors CTI (*F *(1, 42) = 61.4; *p *< .001) and valence (*F *(1, 42) = 9.3; *p *< .01). Given that task-set in PRBs is predetermined (i.e. color in color blocks; shape in shape blocks) and remains constant over the course of 24 trials, task cues and stimulus valence are theoretically irrelevant for performance. Nonetheless, RTs were generally slower on short CTI and bivalent trials. Importantly, a significant main effect for the group factor was again evident in the RT data (*F *(1, 42) = 12.4; *p *< .01; η^2 ^= .228). After excluding the variance in repetition trial performance attributable to the MB context, ADHD group RTs remained significantly slower than control group. While neither CTI nor valence interacted significantly with group (both *F*'s < 1.8), a three-way interaction of these factors was revealed (*F *(1, 42) = 5.7; *p *< .05; η^2 ^= .120). Paired *t*-tests exploring this finding indicated that while ADHD group RTs were significantly slower on short CTI trials comprised of bivalent stimuli than on those with only one task relevant feature (*t*(21) = 3.9; *p *= .001; corrected), valence had no effect on RTs in long CTI trials (*t*(21) = .662; *p *= .5). In contrast, while control group RTs were unaffected by stimulus valence on short CTI trials (*t*(21) = .661; *p *= .5), subjects apparently responded significantly faster on long CTI trials comprised of univalent stimuli than on those comprised of bivalent stimuli (*t*(21) = 3.0; *p *= .007; corrected). Although the discrepancy between control group RTs on long CTI uni- and bivalent trials may have been due to an interference effect, it would be more logical to conclude that it was more likely due to one of two factors: (1) a floor effect or (2) facilitation of RTs on univalent trials. Confirming and extending the group × block type × valence interaction reported in the mixing cost RT analysis, the ADHD group apparently had particular difficulty in maintaining a predetermined task-set when stimuli included distracting features. This interference control impairment was only evident when patient participants did not have time to prepare for the upcoming task.

The respective analysis of the PRB accuracy data revealed no main effects for the CTI or valence factors or an interaction thereof (both *F*s < .54). Task manipulations in PRBs had no general influence on performance accuracy. Indicating that the ADHD committed more errors in PRBs, a significant main effect was revealed for the group factor (*F *(1, 42) = 4.9; *p *< .05; η^2 ^= .140). Specifiying the group × block × CTI finding reported in the presentation of the mixing cost accuracy data, the group factor nearly interacted significantly with CTI in PRBs (*F *(1, 42) = 3.5; *p *< .07) after excluding the variance attributable to MB repetition trial error rates. Despite the marginal significance of this interaction, post hoc independent samples *t*-tests could determine that while group error rates did not clearly differ on short CTI PRBs (*t*(42) = 1.7; *p *= .10), they diverged on long CTI trials (*t*(42) = 3.5; *p *= .001; corrected). However, given the technically insignificant group × CTI interaction, the data should be interpreted accordingly. No further group × condition interactions were witnessed in the PRB block accuracy data (all *F*'s < .7).

## Discussion

Adults with persistent ADHD often attribute their functional impairments to problems such as heightened levels of distractibility and disorganization characterized by frequent switching between uncompleted tasks. Here, we compared the performance of a sample adults diagnosed with childhood-onset ADHD and healthy controls on two experimental measures gauging the efficiency of cognitive control processes in overriding distractability to task-irrelevant features (i.e. Stroop test) and the flexible coordination of multiple task-sets in the face of distraction (i.e. task switching paradigm). In this first study to investigate Stroop interference control in adult ADHD with a trial-by-trial paradigm, group differences were expected to be isolated to measures of interference control. Therefore, control analyses of Stroop test data explored group differences on measures not comprised of distracting task-irrelevant information (i.e. neutral and congruent conditions). Given that no adult ADHD studies have investigated cognitive flexibility with traditional task switching paradigms, we hypothesized group differences on both central cognitive control processes measurable with such tasks: mixing costs (i.e. maintaining task-set) and switch costs (i.e. updating task-set). Specifically, it was expected that the ADHD group would show impaired task-set maintenance and updating as a function of the time allotted to prepare for an upcoming task (i.e. CTI) and the relevance of stimulus features for the two possible tasks (i.e. valence). The specificity of group differences in flexible task-set coordination was controlled for by analyzing performance in blocks where task switching is not required (PRBs).

Evidence for ADHD group impaired interference control was obtained from both tasks. Task switching group error rate profiles revealed distinct cognitive flexibility deficiencies in the ADHD group. Independent of these process-specific group differences, ADHD group performance was found to be generally slower on both tasks. In the case of the task switching paradigm, ADHD group error rates were also found to be generally elevated. In the following, each of these group differences is discussed in turn. We conclude by pointing out limitations of the present study and summarizing possible implications of the findings.

## Group differences in interference control

Perhaps the most striking findings of the current investigation were the ADHD group interference effects on both tasks. Abnormal processing of task-irrelevant stimulus attributes was evident in ADHD group Stroop test performance in RTs and accuracy rates (see again, Fig. 3). Despite generally slower ADHD group Stroop test performance, patient participants were found to be more susceptible to distraction. The specificity of this interference control deficit was further supported by the finding of group performance equivalence as gauged by Stroop facilitation. The degree of response speeding to congruent stimuli was identical for the two groups. Moreover, while the generally slower ADHD group Stroop test performance may have contributed to generally equivalent group error rates, this speed/accuracy trade-off did not aid patient participants in reducing the Stroop effect in error rates.

Two findings from the task switching RT data confirmed that aberrant interference control in the ADHD sample was not merely due to impairment in overriding conflict incited by dominantly represented verbal material. In contrast to the verbal stimuli in the employed Stroop test, the irrelevant dimension of the task switching stimuli on bivalent trials (i.e. color in the shape task/shape in the color task) is not dominantly represented. Given that the ADHD group interference effects on the task switching paradigm were not specifically predicted, they deserve special consideration.

Assuming that ADHD subjects would show more sensitivity to interference on the task switching paradigm when manipulations demanded maintaining or flexibly updating task-set, we hypothesized magnified ADHD group mixing and switch costs as a function of the stimulus valence. However, neither of these effects was observed. Instead, the data surprisingly revealed valence effects in ADHD group PRB performance. First, the group × block type × valence interaction obtained from the mixing cost RT analysis demonstrated that while the control group displayed the expected performance pattern of a larger interference effect in block contexts demanding repeated unpredictable task-set reconfiguration (i.e. MBs), ADHD group performance indicated no such effect. In contrast, while control group RTs did not vary according to stimulus valence in task blocks where task-set is predetermined and does not change on a trial-by-trial basis (i.e. PRBs), ADHD group PRB performance indicated slowing for bivalent vs. univalent trials (see again, Fig. 5). Apparently, ADHD subjects had difficulty extracting stimulus feature relevancy in block contexts demanding cognitive flexibility, but were distracted by task-irrelevant features when context only required selective attention to a singular stimulus feature.

The group × CTI × valence interaction revealed by the RT analysis restricted to PRBs further specified the ADHD group interference control deficit observed on the task switching paradigm. After excluding the variance attributable to MB contexts, the data revealed that the ADHD group interference effect in PRBs was only evident in short CTI trials and could be successfully resolved in long CTI trials. Although task-set is predetermined in PRBs, performance was generally influenced by the CTI manipulation. However, when cues were presented just 100 ms before targets, ADHD subjects could not efficiently allocate selective attention to the relevant stimulus feature. On the whole, the ADHD group interference effects observed in on both tasks deliver strong evidence that interference control is compromised in adults with persisting ADHD. This particular finding of inefficient cue utilization to resolve interference suggests that the interference control impairment in adult ADHD is, at least partially, due to inefficient task-preparation mechanisms.

To our knowledge, only one other study has employed a comparable Stroop task with a ADHD sample. Carter et al. [68] found a group RT interference effect similar to that presented here in a sample of ADHD children performing a trial-by-trial Stroop task demanding verbal responses. However, in contrast to our findings in adults, no general Stroop test performance or interference accuracy differences were observed in the child sample. These differences in findings may be attributable to differing samples and/or response modality. However, given the normal maturation of frontal networks supporting interference control into adulthood (e.g [69]), the possibility that persistence of interference control impairment in ADHD is related to abnormal neurodevelopment within these networks is a hypothesis in need of further investigation. Indeed, evidence for atypical brain development, including frontal regions, in ADHD is accumulating (for a recent review, see [70]). Furthermore, it has been shown that ADHD adults recruited a diffuse frontostriatal network in resolving interference on a blocked Stroop task, while activation patterns in healthy controls were isolated to the anterior cingulate cortex [49].

Several studies have investigated interference control in child and adolescent ADHD with standardized clinical paper and pencil Stroop tasks; culminating in three recent meta-analyses [44-46]. Adult ADHD performance on such traditional Stroop tasks has also recently been quantitatively summarized in two meta-analyses of neuropsychological functioning [42,43]. Although paper and pencil tasks are only moderately comparable to computerized Stroop tasks (see again, [48]), the meta-analytic data suggest that while problems in Stroop interference control remain relatively stable and are at least moderate in magnitude across the lifespan in individuals with ADHD, basic processing speed as measured by rapid color naming (i.e. neutral Stroop trials) appears to become slower as a function of age. Evidence supporting this interpretation of adult ADHD performance on standardized clinical Stroop tests was recently delivered by Gualtieri and Johnson [50]. In a large sample of ADHD individuals and healthy matched controls from age 10 – 29, Gualtieri et al. [50] did not find the degree of significant group interference differences on a computerized blocked Stroop task to covary with age. At the same time, while simple reaction time significantly improved in controls as a function of age, ADHD group basic processing speed was found to stagnate across development. On the whole, despite noteworthy methodological differences, the current findings of impaired Stroop interference control in adult ADHD coupled with generally slower Stroop test performance fit relatively well with previous Stroop/ADHD research. Nonetheless, as suggested the findings of Carter et al [68] and the current investigation, computerized trial-by-trial versions of the Stroop test may be advantageous to more precise investigation of interference control in ADHD.

The findings of larger interference effects in the current sample of ADHD adults on the task switching paradigm correspond only partially with those from previous investigations of explicitly cued task switching in ADHD. In a series of task switching studies incorporating various manipulations of trial timing and S-R compatibility, Cepeda, Cepeda & Kramer [57] and Kramer, Cepeda & Cepeda [58] also observed interference effects in ADHD children. Similar to the valence effects observed here in ADHD adults, Kramer et al. found elevated unmedicated ADHD group error rates on S-R incompatible trails to be unrelated to manipulations targeting switching processes. However, Cepeda et al. [57] found group interference effects in RTs to be specific to switching. A comparison of the childhood ADHD interference effects found with task switching paradigms and the data presented here suggests that impaired interference control is not task-specific and remains significant in adult manifestation of the disorder.

## Group differences in cognitive flexibility

Contrary to our hypotheses, we did not observe group switch or mixing cost RT differences in task switching performance. The relative magnitude of RT differences between MB switch vs. repetition trails, as well as between MB repetition and PRB trials was identical for the two groups. While the group mixing cost RT equivalence replicates previous findings in children, the group switch cost RT equivalence stands in contrast to the central findings of both previous cued task switching/ADHD studies [57,58]. However, as discussed below, two group error rate differences obtained with the task switching paradigm suggest that inefficiency of task-set coordination persists in adults ADHD.

As demonstrated by the three-way group × trial type × CTI interaction in the MB accuracy data, an elongated preparatory interval was insufficient for ADHD subjects to flexibly update task-set (see again, Fig. 4). While the control group was able to reduce error rate switch costs on long CTI trials, ADHD group switch costs were not influenced by the CTI manipulation. This finding suggests impaired transient task-set reconfiguration in adult ADHD. In contrast, the three-way group × block type × CTI interaction revealed by the mixing cost analysis of the accuracy data indicated inefficient sustained task-set maintenance in the ADHD group (see again, Fig. 6). Despite the difficult interpretability of the atypical control group response accuracy pattern, ADHD group mixing costs were evident on short CTI trials. However, following long preparation intervals ADHD subjects were clearly able to eliminate costs on repetition trials in MBs in comparison to those making up PRBs. ADHD group advance preparation was successful in reducing mixing costs but not switch costs. Taken together, these two findings shed light on the nature of the cognitive control impairment in adult ADHD. As measured by the employed task switching paradigm, our ADHD sample apparently had much less of a problem holding task-sets online in MB vs. PRB contexts than flexibly switching between two task-sets.

A further group effect which approached significance (group × CTI in PRB error rates) suggests that the above described group switching differences (both group effects were interactions with the CTI factor) may be attributable to a general ADHD group deficiency in task preparation mechanisms. Importantly, a lifespan study of task switching abilities in healthy individuals found all investigated age groups to benefit equally from advance preparation [71]. Suggestive of intact task preparation in childhood ADHD, Kramer et al. [58] did not find any group differences to be attributable to the CTI manipulation. Although it is possible that our findings of inefficient preparatory mechanisms in ADHD adults may be a developmental consequence of the disorder, differences in findings may also be attributable to differing task and/or sample characteristics. Nonetheless, ADHD group task switching performance appeared to be particularly influenced by the amount of time allotted to prepare for an upcoming task. Taken together, the observed deficits in keeping task-sets online and flexibly switching between task-sets may not be due to top-down control failure, but rather ineffective bottom-up signaling for the need for control.

## General group performance differences

Clinical group performance on cognitive tasks is commonly found to be *generally *suboptimal. Indeed, ADHD participants were found to produce generally slower responses under all conditions of both of the employed tasks. ADHD group task switching error rates were also found to be generally elevated. Given this fundamental difference between clinical and control groups, researchers tend to ignore general group differences and only interpret group × condition interactions. However, it is becoming increasingly recognized that generalized performance deficits may be particularly informative (for a recent review, see [72]). In the case of ADHD, studies analyzing performance distributions [73-77], see also [78]) have revealed that generalized slowing can be largely attributed to intra-individual response variability including a disproportionate number of abnormally slow RTs. It has even been suggested that response inconsistency may be more of a defining neuropsychological characteristic of ADHD than impairment in specific executive domains (see again, [9]). A valid fine-grained performance distribution analysis of the current data set was prevented by the relatively small number of trials making up the employed paradigms. Nevertheless, as discussed below, the data provide an informative basis for the interpretation of the observed ADHD group generalized slowing.

Recent meta-analyses of neuropsychological functioning in adult ADHD have delivered differing interpretations for the relationship between generalized slowing and poor process-specific performance on a variety of tasks [42,43]. Boonstra et al. [43] commented that group differences found on measures of executive functioning are plausibly due to generalized slowing. In contrast, Hervey et al. [42] suggested that generalized performance impairment in adult ADHD may be partially explained by inefficient allocation of effortful attention. More in accordance with the latter view, particularly the findings of ADHD group interference effects in task switching PRBs suggest that the simultaneously observed generalized slowing may be a by-product of inefficient cognitive control implementation. ADHD subjects apparently executed cognitive control continuously, as opposed to selectively when necessary. To illustrate, in task blocks in which subjects were required to classify stimuli according to color in each of the 24 trials (blue or red), ADHD group performance was influenced by stimulus shape. In shape task blocks, ADHD subjects apparently attended to stimulus color. These findings demonstrate that ADHD subjects were superfluously executing cognitive control in task contexts requiring selective attention to a singular feature. This inefficient allocation of cognitive control may have contributed to generalized slowing.

Still yet, it cannot be ruled out that the generalized ADHD group performance impairment was due to response inconsistency. As mentioned, a valid fine-grained performance variability analysis would not make sense with the relatively few trials making up the employed tasks. However, as a crude measure of intra-individual response speed variability, we calculated coefficients of variation (RT^SD^/RT^MEAN^) for each subject under all conditions of both tasks and for the complete experiments [79]. The only noteworthy group difference in the individually determined coefficients of variation was found in PRB performance (*t*(42) = 2.5; *p *= .015) (all other *t*'s < 1.4). Although this effect would not have survived Bonferroni correction, ADHD group RTs appeared to fluctuate to a greater degree than those of the control group only in simple task contexts where demands do not unpredictably change on a trial-by-trial basis. Not incidentally, ADHD group interference effects in the task switching paradigm were also observed in PRB's. An important matter of future neuropsychological research of ADHD should be to clarify whether performance inconsistency can explain not only general performance deficits, but also those cognitive control impairments currently thought to be at the core of the disorder.

Another particular alternative interpretation of the generally deficient ADHD group performance is entirely plausible and warrants special mention. Currently accumulating findings suggest that abnormally slow ADHD group performance on tasks comprised of colored stimuli are possibly due to hypofunctioning retinal dopaminergic transmission [80,81]. Given that all target stimuli in the employed paradigms were presented in color and possibly contributed to the overall slowing of the ADHD group, future studies should employ tasks in which the variable of color perception is controlled.

## Limitations

Aside from the possibly confounding factor of color stimuli and the relatively short duration of the employed paradigms, notable limitations of the current study include the small patient sample of convenience and a failure to include manipulations allowing for more direct testing of dual-process model hypotheses. Given that these shortcomings may have an influence on interpretability, readers are suggested to heed caution when drawing their own conclusions.

Regarding the sample, patient participants represented only a subgroup of individuals with persisting ADHD (see e.g. [82]). On the whole, patients had average to above-average IQ and were, despite significant adaptive impairments, relatively high-functioning, educated, and employed/employable individuals. Future studies should attempt to incorporate individuals not actively seeking treatment. Although the gender ratio in our patient sample corresponds with childhood ADHD epidemiological data, it diverges from adult ADHD prevalence rates. However, given that we excluded individuals reporting more significantly impairing comorbid disorders and findings that adult females with ADHD have higher rates of clinically relevant comorbid anxiety and mood disorders [83], we believe the underrepresentation of females in the sample mirrors the high-functioning, relatively comorbity-free adult ADHD population fairly well.

Patient participants were diagnosed with "ADHD in partial remission" [1] instead of specifying subtypes due to the lack of age-appropriate diagnostic criteria for ADHD in adulthood. Optimally, ADHD subtypes for adults would be determined according to the persistence/transience of childhood subtypes, as has been recently done [84]. Given the size of our sample, sufficient statistical power for an analysis of the present data set regressed onto subtype would not have been achieved. Unfortunately, this imperfection has the consequence that we cannot discern whether the observed group effects were general or attributable only to a certain ADHD subtype.

Regarding the tasks, the employed paradigms did not include properties which allowed for precise direct measurement of dual-process model hypotheses of inefficient bottom-up state regulation policies in ADHD. Although some evidence of inefficient bottom-up engagement of cognitive control in the current ADHD sample was obtained (e.g. interference effects in PRB's, inefficient task preparation as evidenced by multiple group × CTI interactions), task manipulations would not allow for definitive conclusions in this respect. In order to assess the applicability of child-based neuropsychological models of ADHD to adult manifestation of the disorder, future studies of cognitive control should employ paradigms which manipulate factors such as event rate or reward. If dual-process models are to be extended to apply to adult ADHD, deficits in cognitive control should be dependent on factors directly tapping bottom-up mechanisms.

## Conclusion

The current study illuminates the utility of employing experimental tasks borrowed from the basic cognitive sciences to investigate processes hypothesized to be impaired in a neurocognitive disorder. Although contemporary neuropsychological models of ADHD predict interference control and cognitive flexibility deficiencies in the disorder, previous findings obtained with traditional measures (e.g. paper and pencil Stroop tests, WCST) have been inconsistent. Here, we found consistent group interference control differences on two independent tasks. Experimental manipulation of the time allotted to prepare for an upcoming task revealed that the ADHD group interference effect observed on the task switching paradigm was dependent on inefficent task preparation. In fact, all switching-related group differences were also found to be dependent on atypical preparation effects. While ADHD group advance task preparation processes were efficient enough to maintain task-sets online in the context of repeated unpredictable task switching, it failed when transient task-set updating was demanded. Aside from these process-specific group differences, ADHD group performance was also generally slower and less accurate. Although not a central question of the current investigation, anecdotal evidence demonstrated that intra-individual response variability may have accounted for generalized ADHD group response slowing. However, this possibility was only evident in relatively undemanding pure repetition blocks of the task switching paradigm – exactly those task contexts in which ADHD group interference effects were also observed. Taken together, ADHD group deficits in interference control and cognitive flexibility could not be clearly dissociated from abnormal preparatory mechanisms and/or response inconsistency. Thus, it remains inconclusive as to whether ineffecient ADHD group cognitive control was due to top-down failure or bottom-up engagement thereof. To clarify this issue, future neuropsychological investigations are encouraged to employ tasks with significantly more trials and direct manipulations of bottom-up mechanisms with larger samples.

## Competing interests

The author(s) declare that they have no competing interests.

## Authors' contributions

JAK conducted data collection, data analysis, literature review and prepared all drafts of the manuscript; MC examined all subjects and made all final diagnostic judgments; MB programmed experiments and significantly contributed to draft revision; DYC instigated supplementary analyses and contributed substantially to theoretical interpretation; IH instigated cooperation between the participating institutes. All authors have read and approved the final manuscript.
